# Dental Treatment at Tuberculosis Dispensaries

**Published:** 1914-01-24

**Authors:** 


### DENTAL TREATMENT AT TUBERCULOSIS DISPENSARIES.

By A TUBERCULOSIS DISPENSARY OFFICER.

A tuberculosis dispensary has admittedly two sets of functions, the preventive and the curative, though the latter may perhaps better be described as ameliorative under present conditions. As regards either of these functions, a tuberculosis dispensary cannot stand alone. That is to say, the equipment of a dispensary does not comprise merely a doctor with a stethoscope and a nurse with a visiting list, even with a drug-store and a tuberculin syringe thrown in. Not only is close co-operation desirable with the public health authorities, but ready access is necessary to the special departments of a hospital. Better still is it to have two at least of these special departments on the spot—namely, the radiographic and the dental department. The help in diagnosis which can be rendered by the *x*-rays is now fully recognised in tuberculosis of the lungs, but not so the importance of dental treatment for the phthisical.

This neglect of oral hygiene is all the more surprising when it is remembered how universally it is agreed that the most effective method of combating the disease is by maintaining the patient's nutrition at as high a level as possible. To this end it is customary to give away to phthisical patients enormous quantities of stomachic medicines of the type of mist. gent. alk. Day after day this goes on in every hospital and dispensary in the kingdom, streams of tonic bitters being dispersed by hard-worked dispensers for the sole benefit, in the vast majority of cases, of the wholesale druggists.

It is scarcely necessary to labour the point that the presence of oral sepsis is widespread amongst the phthisical. When dental caries is admitted to be present to such a fearful extent as is disclosed by the results of school inspection, no one will doubt the prevalence of oral sepsis and caries in the patients attending a tuberculosis dispensary. Not only is it easy for anyone to satisfy himself on this point, but it is almost equally easy for him to be convinced of the enormous change for the better in such patients as have had their mouths put in order.

### An Object-lesson.

An object-lesson of this sort will not soon be forgotten, and fortunately such cases are to be observed at some tuberculosis centres thanks to the co-operation of some painstaking dental surgeon interested in tuberculosis work. But until an observer has had the opportunity of looking on this picture and on that—the " before " and the " after " —he is perhaps inclined to remain sceptical. The large series of cases reported by Dr. William Hunter contain several in which the relation of cause and effect was beyond cavil, and in which the results of treatment were little short of marvellous. In some of these there was an absence of any gross sepsis in the mouth, and very careful search was required to find the mischief, yet there was no doubt that even so comparatively limited a focus was the actual cause of severe constitutional symptoms. These cases have no direct bearing on the cases to be seen in a tuberculosis dispensary, but serve to show that the connection between oral sepsis and ill-health is no theorem or enthusiast's fad.

How, then, can it be considered justifiable to attempt to treat consumptives without proper attention being paid to the one region of all others through which the nutrition of the patient can be most beneficially affected? Of all the general methods of treatment it is safe to say that none is so productive of good result as the elimination of oral sepsis in its widest sense. Common sense tells us that this must be so, for it is admitted that all the measures at present employed against pulmonary tuberculosis have for their objective the increasing of the resistance of the patient. Sanatorium treatment and drug treatment are but indirect methods, and if dental treatment is also indirect it is surely less so, inasmuch as it directly influences the assimilation of food. It is no argument against the maleficence of oral sepsis to say that many persons have a gross degree of sepsis in their mouths and yet do not suffer from gastritis or anæmia. Such a semblance of good health is apparent only, and a definite improvement in general well-being and assimilation will always follow the abolition of the toxin-producing stomatitis. Within the past twelve months the writer has seen three cases notified as typhoid fever in which the fever and the enteritis were solely due to the septic foci in the mouth. In one case the mouth was apparently in good order, but contained several gold crowns and some bridge work—structures which have been aptly described by Hunter as a " mausoleum of gold," and which he has shown to be absolutely incompatible with a healthy mouth. Nothing may be noticeable for a time, but as surely as time flies Nemesis in the guise of anorexia, indigestion, or a sallow complexion will follow. Often a slight degree of salivation, constant and annoying, is all that is noticed for some time.

In dispensary practice it is, of course, not a question of gold crowns and bridge work, but a matter of fillings and extractions. The exigencies of the circumstances may only allow extractions and the attacking of the gingivitis, but even this will be followed by extraordinary improvement. It should not be forgotten that before extractions are done the gums in septic cases require assiduous antiseptic treatment for a few days, lest the wounds made by the extractions allow septic infection to spread. In some cases it will be found that a patient with edentulous gums will get on far better than one might expect, and a thousand times better than he or she did with the tender, septic, and useless remains of teeth. In any case sepsis must not be allowed to wait the convenience or opportunity (pecuniary or otherwise) of a denture to replace the offending stumps. The tuberculosis dispensary must step in and regard dental treatment as a part of its routine, like dispensing or auscultation.

